# Diapause in a tropical oil-collecting bee: molecular basis unveiled by RNA-Seq

**DOI:** 10.1186/s12864-018-4694-x

**Published:** 2018-04-27

**Authors:** Priscila Karla F. Santos, Natalia de Souza Araujo, Elaine Françoso, Alexandre Rizzo Zuntini, Maria Cristina Arias

**Affiliations:** 1 Departamento de Genética e Biologia Evolutiva, Instituto de Biociências, Universidade de São Paulo, Rua do Matão, 277, Room 320, São Paulo, SP CEP 05508-090 Brazil; 2 Current address: GIGA – Medical Genomics, Unit of Animal Genomics, University of Liege, Quartier Hopital, Avenue de I’Hopital, 11, 4000 Liege, Belgium; 3 Departamento de Biologia Vegetal, Instituto de Biologia, Universidade Estadual de Campinas, Rua Monteiro Lobato 255, Barão Geraldo, Campinas, SP CEP 13083-970 Brazil

**Keywords:** *Tetrapedia diversipes*, Solitary bees, Transcriptome

## Abstract

**Background:**

Diapause is a natural phenomenon characterized by an arrest in development that ensures the survival of organisms under extreme environmental conditions. The process has been well documented in arthropods. However, its molecular basis has been mainly studied in species from temperate zones, leaving a knowledge gap of this phenomenon in tropical species. In the present study, the Neotropical and solitary bee *Tetrapedia diversipes* was employed as a model for investigating diapause in species from tropical zones. Being a bivoltine insect, *Tetrapedia diversipes* produce two generations of offspring per year. The first generation, normally born during the wet season, develops faster than individuals from the second generation, born after the dry season. Furthermore, it has been shown that the development of the progeny, of the second generation, is halted at the 5th larval instar, and remains in larval diapause during the dry season. Towards the goal of gaining a better understanding of the diapause phenomenon we compared the global gene expression pattern, in larvae, from both reproductive generations and during diapause. The results demonstrate that there are similarities in the observed gene expression patterns to those already described for temperate climate models, and also identify diapause-related genes that have not been previously reported in the literature.

**Results:**

The RNA-Seq analysis identified 2275 differentially expressed transcripts, of which 1167 were annotated. Of these genes, during diapause, 352 were upregulated and 815 were downregulated. According to their biological functions, these genes were categorized into the following groups: cellular detoxification, cytoskeleton, cuticle, sterol and lipid metabolism, cell cycle, heat shock proteins, immune response, circadian clock, and epigenetic control.

**Conclusion:**

Many of the identified genes have already been described as being related to diapause; however, new genes were discovered, for the first time, in this study. Among those, we highlight: *Niemann-Pick type C1*, *NPC2* and *Acyl-CoA binding protein homolog* (all involved in ecdysteroid synthesis); *RhoBTB2* and *SASH1* (associated with cell cycle regulation) and *Histone acetyltransferase KAT7* (related to epigenetic transcriptional regulation). The results presented here add important findings to the understanding of diapause in tropical species, thus increasing the comprehension of diapause-related molecular mechanisms.

**Electronic supplementary material:**

The online version of this article (10.1186/s12864-018-4694-x) contains supplementary material, which is available to authorized users.

## Background

Diapause is a temporary state of developmental arrest that is hormonally programmed in advance of environmental adversities, such as food restriction, stress and temperature variation. The developmental halt is only broken when environmental conditions become favorable again, however this return does not occur immediately [1, 2]. Photoperiod is the most well understood diapause stimuli, with pronounced effects on many organisms in temperate zones [3, 4]. In tropical species, besides photoperiod, other environmental signals, such as changes in nutrition quality, humidity, and rainfall are also known to induce diapause [3].

In insects, diapause may occur at any developmental stage (embryo, larva, pupa or adult) [5], and varies from a decrease in activity (i.e. reproductive diapause) to complete developmental arrest (i.e. developmental diapause) [6]. Diapause normally consists of three phases: pre-diapause, diapause, and post-diapause [4], and the phase transitions are mediated by molecular mechanisms guided by variations in gene expression [7]. It is known that some genes are silenced, whereas others are exclusively expressed during specific phases [5]; however, the exact level of evolutionary overlap among molecular networks underlying this phenomenon is still an open field for investigation [7].

Despite the identification of some unique and controversial patterns of gene expression in a number of organisms, the existence of an insect diapause genetic toolkit has been suggested [8]. According to this hypothesis, some genes would be expressed similarly in diapause, thus regulating common physiological processes across insect taxa [9]. For example, a diapause study, in the Hymenoptera *Bombus terrestris*, revealed several genes that showed a common expression pattern during diapause in the Diptera *Sarcophaga crassipalpis*. Most of these genes were related to insulin, juvenile hormone, nutrient storage, and stress resistance pathways [10]. Furthermore, a recent study, comparing diapause transcriptomic data from multiple insect groups, showed that the gene expression pattern, among species, clustered according to the stage of diapause occurrence instead of by the phylogenetic relationships of the insects, thus further corroborating the toolkit hypothesis [9].

*Tetrapedia diversipes,* an oil-collecting solitary bee endemic of the Neotropical region [11], is a promising model for studying molecular changes related to diapause. This species is bivoltine, producing two generations per year. The first generation (G1) is born in nests, built by adult females, during late spring/early summer (wet season). While the second generation (G2), emerges from nests built during late summer/early fall (dry season) [12]. The total developmental time (i.e. from egg to adult) for G1 is shorter (74.4 days on average) than that observed for G2 (224.6 days on average) [13]. Previous studies have shown that in G2, mature 5th instar larvae remain inactive and in diapause, through the months of July and August (dry season), and that nesting activities are also drastically reduced during this period [14]. Although, the exact environmental cue that initiates diapause in *T. diversipes* is still unknown, previous studies have reported that there are substantial changes in the floral resources used by this bee, depending on the season [15, 16]. These differences in food provisioning may be the environmental stimulus responsible for the diapause response, but this is still speculative.

In addition to bivoltinism and diapause, *T. diversipes* also display a high nesting rate in trap nests [13, 17], which enables researchers to monitor development and sample specimens under natural conditions. This provides an excellent opportunity to investigate naturally occurring diapause, since there is no need to induce this process under laboratory conditions and, moreover, one generation can be used as the control for the other.

In this context, the aim of the present study was to investigate differentially expressed genes (DEGs) involved in the diapause of *T. diversipes* and comparatively analyze these findings. RNA-Seq data of non-diapause larvae from both generations were compared to diapause larvae data, and DEGs were correlated with previous studies to identify conserved patterns among organisms.

## Methods

### Sample collection

Experiments were conducted at the University of São Paulo campus (São Paulo, Brazil). The city of São Paulo is situated in a border region of tropical and subtropical climates at latitude 23°S, thus environmental differences between the dry and wet seasons are not as intense as in temperate climates. In this area, lower levels of temperature and precipitation (i.e. dry season) are reported from April to September, whereas the lowest marks are observed from July to September (http://www.estacao.iag.usp.br/Boletins/2013.pdf) [18].

The trap nests were built according to [13] and placed in a garden area of the campus (23°33’S). *Tetrapedia diversipes* larvae of all instars from the first generation (nests marked and collected in November and December) and second generation (nests marked in March and April and collected from March to July), were collected from several trap nests between 10:00 A.M. and 12:00 P.M., immediately frozen in liquid nitrogen, and stored at − 80 °C. The 5th instar larvae in diapause were collected at the end of July and August (nests marked in March / April), and sampled under the same conditions (sampling methods were based on the observations of [13, 14]).

### RNA extraction and sequencing

The RNA was extracted from each individual larva using the RNeasy Mini Kit (Quiagen, Austin, Texas, USA), following quantification in an EpochTM Spectrophotometer System (BioTek, Winooski, Vermont, USA). Three larvae were pooled per sample (2 μg of total RNA from each larva) to normalize individual differences. Three replicate samples of each state: G1 non-diapause; G2 non-diapause and G2 diapause were prepared, thus totalizing nine sequenced samples (Table 1). This pooling strategy was intentionally used to broadly investigate differences in gene expression among G1 and G2 whole larval development and G2 diapause larvae. Raw RNA-Seq data for non-diapause larvae (G1 and G2) were previously generated and reported by [19].

**Table 1:** The nine samples (pools of three larvae) names and details concerning larval instars, status (non-diapause and diapause) and generation (1st or 2nd)

Status	Generation	Sample Name^a^	Instars	Collection date
Non-diapause	1st	L1	1st - 4th	Dec/2012
		L2	2nd - 5th	Nov/2012
		L3	5th	Nov-Dec/2012
	2nd	L4	5th	Mar-Apr/2013
		L5	1st - 4th	Apr/2013
		L6	2nd - 5th	Mar-Apr-Jul/2013
Diapause	2nd	D1	5th	Jul/2013
		D2	5th	Aug/2013
		D3	5th	Aug/2013

^a^Three larvae pool

Prior to sequencing, RNA quality was measured for each sample using an Agilent 2100 Bioanalyzer®. One library per sample was prepared using the Illumina® TruSeqTM RNA Sample Preparation Kit (Illumina, San Diego, California, USA), and paired end reads of 100 bp were sequenced using the Illumina® HiSeq 2000 platform. These procedures were performed by Macrogen (South Korea).

### Cleaning and assembly

The sequencing quality was visualized in FASTQC-0.10.1 (http://www.bioinformatics.babraham.ac.uk/projects/fastqc/). To eliminate errors, due to bias in library construction [20], the first 14 bases of each read were removed using FASTX-0.0.13 (http://hannonlab.cshl.edu/fastx_toolkit/index.html). Bases of low quality, as indicated by a phred score of < 30 [21], and reads shorter than 31 bases were removed using SeqYclean-1.8.10 (https://github.com/ibest/seqyclean).

The Trinity-2.0.6 program [22] was used for digital normalization and assembly. Reads from all libraries were concatenated to build the whole transcriptome of *T. diversipes*. The default parameters were utilized, with the exception of the minimal kmer coverage (−min_kmer_cov 10) and the minimum number to glue contigs together (−min_glue 30). Analyses were performed using the cloud computing service at the University of São Paulo (USP) and at the Laboratory of Genetics and Evolution of Bees (LGEA) - USP. Transcriptome assembly quality was evaluated using the Benchmarking Universal Single-Copy Orthologs (BUSCO) software [23].

### Annotation and gene expression analysis

Gene annotation was performed by Blastx [24] in the Annocript-1.1 software [25]. The UniRef databases, from September of 2015, were used and results from both UniRef [26] and Swiss-Prot [27] were considered (*p*-value 1e-5). In the case of divergence between these two databases, the Swiss-Prot output prevailed since this database is manually curated and is therefore considered more reliable [28]. Transcripts annotated as potential contaminants (acari, bacteria, fungi, virus and plants) were removed according to [15].

For differential gene expression analyses, reads from each cleaned library were aligned to the final *T. diversipes* transcriptome using Bowtie2–2.2.5 [29] and counted with RSEM-1.2.22 [30]. Significant DEGs were identified with DESeq2 [31]. For these statistical analyses two groups were compared: non-diapause larvae (G1 and G2 samples – 6 replicates) and diapause larvae (3 replicates). Statistical test of significance (minimum FDR *p*-value <1e-5) was performed. The data analysis and heat map generation were automated using the Trinity-2.0.6 scripts according to the default parameters. The Blast2GO software [32] was used to perform the Gene Ontology (GO) term enrichment analysis using the one-tailed Fisher’s exact test, using the default parameters.

## Results and discussion

### Transcriptome, DEG and GO analysis

#### Cleaning, assembly, and annotation

The raw RNA-Seq data resulted in 534,586,824 reads. After the cleaning and digital normalization steps, the read number decreased to 464,537,144 (86.9%) and 29,650,152 (6.4%), respectively. The final data set was concatenated and used for de novo assembly, which resulted in 29,320 contigs with a N50 of 1781 bp and a contig median length of 580 bp. By comparing these sequences to the sequences in the Hymenoptera database, the BUSCO program identified 3656 (82.8%) transcripts as complete single-copy genes but 367 (8.3%) genes were not found in the assembled transcriptome.

The annotation procedure, performed with Annocript, identified 16,237 transcripts as potential coding genes, corresponding to 55.4% of the complete transcriptome assembled. A total of 8928 sequences were annotated using the Swiss-Prot database and 13,223 using UniRef. Additionally, 76 transcripts corresponded to ribosomal RNA and 892 to potential long non-coding RNAs. Thus, 17,205 (58.6%) of the assembled transcripts had their putative function determined. From the UniRef annotated genes, 2296 presented high homology to sequences from *Bombus*, followed by *Apis* (1667), and *Megachile* (1390). It is also worth mentioning that 394 transcripts of contaminants, including plants, were identified and excluded.

#### Differentially expressed genes

**Fig. 1:**
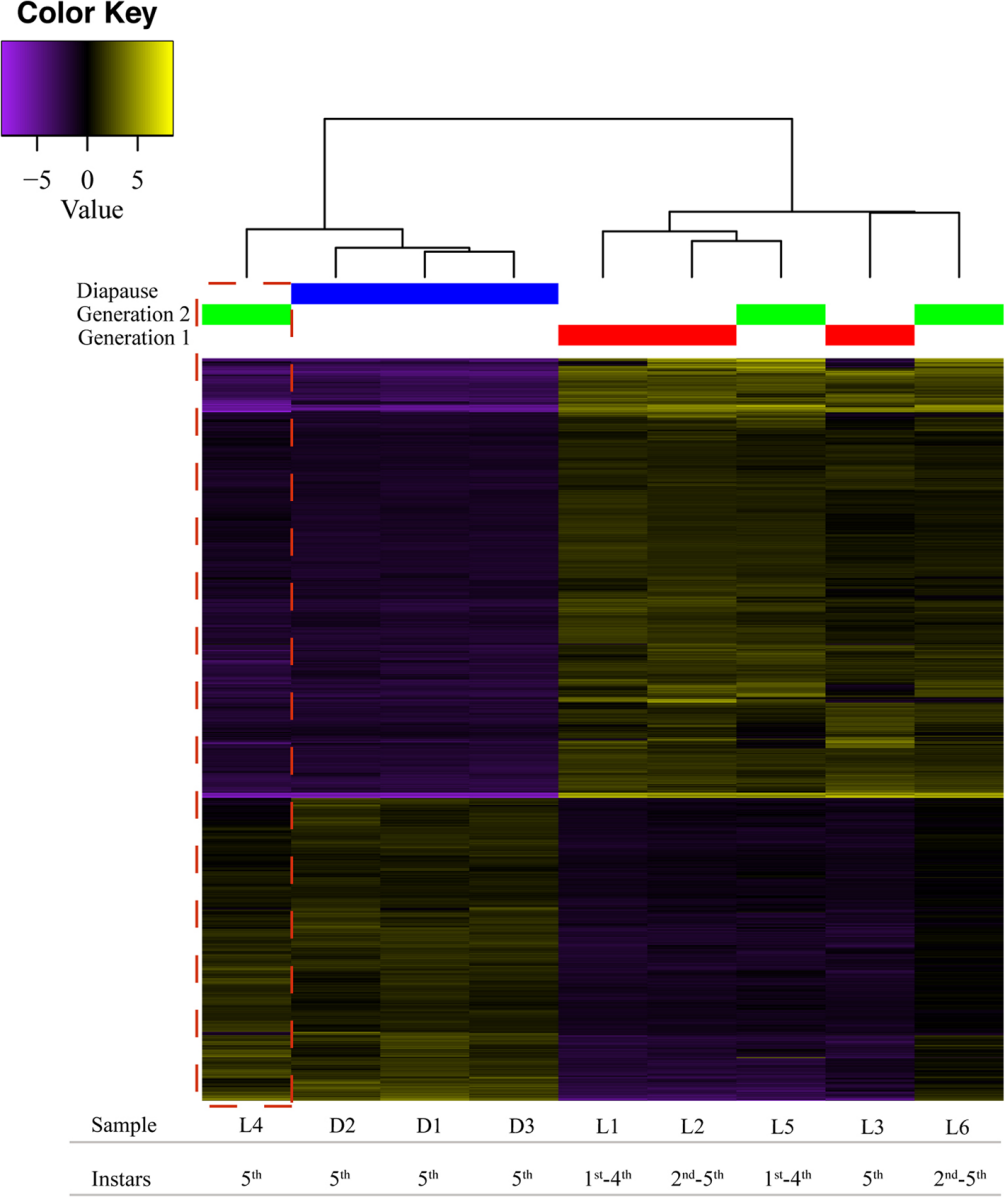
Heat map of the 2274 transcripts identified as differentially expressed (between diapause and non-diapause larvae) by DESeq2. The yellow and purple colours indicate high and low expression, respectively. Each column represents the transcripts of one sample (as indicated in Table 1) (L1 to L6: non-diapause larvae; D1 to D3: diapause larvae), and each line corresponds to a differentially represented transcript. The larvae instars are indicated for each sample. Generation 1, 2, and Diapause are represented by red, green, and blue bars on the top, respectively. The dashed red line highlights the non-diapause sample, from the second generation, with a gene expression pattern similar to diapause samples. Expression value scale is on log2

DESeq2 identified 2274 differentially expressed transcripts (Fig. 1), of which 1167 (51.3%) could be annotated. From these, 352 were upregulated and 815 were downregulated in diapause larvae (Additional file 1: Table S1 and Additional file 1: Table S2, respectively). This trend in gene expression is consistent with previous studies that showed, during insect diapause, genes are expected to be more downregulated than upregulated [5].

As described in the Methods section, different larval instars were pooled, and represented the non-diapause groups (from G1 and G2), while the diapause group contained only 5th instar larvae (from G2) collected late in the dry season. It should be mentioned that, besides exclusive 5th larvae instar replicates, other non-diapause replicates also encompass this developmental stage (i.e. G1 and G2: L2, L3, L4 and L6), thus adding specific gene expression variation of the 5th instar larvae to the sample. By using this approach, we intended to reduce the misidentification of genes related exclusively to instar differences. Additionally we have adopted a very conservative *p*-value as the threshold for identifying DEGs. As indicated in Fig. 1, these methods successfully grouped all non-diapause samples regardless the larval instars, with the exception of L4. This suggests that specific gene expression differences were normalized as desired. Therefore, the results discussed herein should be considered as a broad analysis of genes involved in *T. diversipes* diapause.

The L4 sample contained 5th instar larvae from G2 with a unique profile of gene expression, as evidenced by an intermediate gene expression pattern between diapause and non-diapause libraries (Fig. 1), which is also evident in the normalized counts matrix generated by DESeq2 (Additional file 1: Table S3). These larvae were collected early in the second reproductive season, before the apex of environmental changes (Table 1). Therefore, it is plausible that the L4 sample represents either the beginning of diapause (the pre-diapause phase) or that some of the pooled larvae in this sample were already in diapause, since the larvae may enter diapause before extreme environmental conditions are reached [13]. More detailed studies are still needed to better understand this observed pattern. Interestingly, this sample had a different profile from the 5th instar larvae from G1, thus providing additional evidence that the major differences observed in the present DEG analyses are due to the diapause state and are not the result of development.

#### Gene ontology terms enrichment

In the GO term enrichment analysis, no enriched term was identified among the upregulated genes. Nonetheless, over-represented GO categories were verified in the analysis of 377 downregulated genes (Additional file 1: Table S4). This result may be due to a bias in the annotation process, since the upregulated genes seem to be under represented in the databases, and as a result failed to be annotated (Additional file 1: Figure S5). Whether the non-annotated transcripts are genes specifically related to diapause, but not yet characterized because most of the data are from model organisms, is a matter for future research. Furthermore, the databases need to be populated with data from non-model organisms.

With regards to over-represented GOs, the tricarboxylic acid (TCA) cycle term stood out. The TCA cycle is also suppressed during pupal diapause in *Sarcophaga crassipalpis* [33] and *Helicoverpa armigera* [34]. Its inhibition has an important role in energy conservation during diapause [34]. In fact, this cycle has been referred to as a checkpoint for regulating different forms of dormancy. This is because low TCA activity decreases ecdysteroid levels, resulting from a downregulation of prothoracicotropic hormone, and as a consequence ceases the synthesis of ecdysone, which maintains the state of developmental arrest [35].

### Genes and diapause

Diapause is a dynamic process marked by physiological transitions. Physiological changes occur due to alterations in central metabolic pathways, such as insulin signaling, lipid storage, stress responses, cell cycle, as well as others [5, 10, 36], and are driven by the regulation of gene expression. Nonetheless several studies seeking for a conserved genetic toolkit have failed to identify a pattern. This is due to the fact that expression levels of the genes are highly variable, and independent of the species and developmental phase at which diapause occurs (Table 2). For example, the genes *phosphoenolpyruvate carboxykinase* and *proliferating cell nuclear antigen* are suggested to be components of the insect diapause genetic toolkit [8] however, their expression levels do not change in *T. diversipes* non-diapause or diapause larvae, nor in *Megachile rotundata* diapause or post-diapause larvae [37].

**Table 2:** Comparative table indicating some examples of differentially expressed genes and genes related to the categories tubulin, actin, myosin, and cuticle proteins that present a different pattern of expression among the species. For each species, the development phase in which diapause occurs and whether the gene or protein is upregulated (↑) or downregulated (↓) is indicated

Species	Phase	Genes							Categories			
		*Gst*	*UGT*	*ACBP*	*NPC1*	*Pcna*	*Pepck*	*Titin*	Myosin	Tubulin	Actin	Cuticule
*Tetrapedia divesipes*	Larva	↓	↓	↓	↓			↑	↑↓	↑	↑	↓
*Caenhorhabditis elegans*	Larva		↑^1^		↓^2^	↓^3^	↑^4^					
*Ostrinia nubilalis*	Larva	↓^5^										
*Chymomyza costata*	Larva	↑^6^	↑^6^			↓^7^						
*Aedes albopictus*	Larva pharato					↓^8^	↑^8^					↑^8^
*Megachile rotuntada*	Prepupa	↓^9^			↓^9^				↓^9^	↑↓^9^	↑↓^9^	↓^9^
*Helicoverpa armigera*	Pupa	↑^10,11^		↓^12^						↑^13^	↑↓^14^	↑^10^
*Sarcophaga crassipalpis*	Pupa					↓^18^	↑^19^					
*Culex pipiens*	Adult										↑^20^↓^21^	↑^21^
*Tetranychus urticae*	Adult	↑↓^3^				↓^3^	↑^21^	↑^21^	↑^21^	↑^21^	↑^21^↓^15^	
*Bombyx mori*	Egg		↑^16^					↑^17^			↑^17^	↓^16^

References: ^1^ [96], ^2^ [97], ^3^ [39], ^4^ [98], ^5^ [99], ^6^ [100], ^7^ [36], ^8^ [8], ^9^ [37], ^10^ [46], ^11^ [34], ^12^ [49], ^13^ [73], ^14^ [101], ^15^ [68], ^16^ [102], ^17^ [103], ^18^ [104], ^19^ [33], ^20^ [44], ^21^ [105]

To contribute to the identification of genes possibly involved in the insect diapause genetic toolkit, we have structured the following discussion based on genes previously described in this process. In addition, some new genes with the potential of being included in the genetic toolkit are also discussed. In the following discussion, genes are organized into categories consonant to their function.

#### Cellular detoxification

*Glutathione-S-transferase* (*GST*) and *UDP-glucuronosyltransferase*, genes related to cellular detoxification, were downregulated during *T. diversipes* and *Ostrinia nubilalis* (Lepidoptera) larval diapause [38], as well as in the reproductive diapause of *Tetranychus urticae* (Arachnida) [39]. GST adds a glutathione group to xenobiotics and its derivatives, which allows these compounds to be catalyzed by enzymes, thereby preventing them from interacting with cellular components [40]. On the other hand, UDP-glucuronosyltransferase participates in cellular detoxification through a different mechanism. It adds a glucuronic acid to lipophilic molecules, thus increasing their water solubility [41]. In *O. nubilalis* and *T. urticae*, it is suggested that the downregulation of *GST* may be associated with non-feeding, during diapause, since this behavior will also reduce xenobiotic ingestion [38, 39]. The same reasoning could also be used to explain the observed downregulation of this gene during *T. diversipes* diapause.

#### Cytoskeleton and cuticle

Different isoforms of *actin*, *myosin*, and *tubulin* genes were upregulated during *T. diversipes* diapause. Myosin and actin proteins interact to generate the contractile strength of muscles, and actin and tubulin comprise the microtubules (filaments of the cytoskeleton) [42]. Cytoskeleton components were suggested to contribute to the survival of an organism during the cold season [43]. Actin genes were also increasingly expressed in diapause of *Culex pipiens*, presumably in response to low temperatures [44]. Different isoforms of myosin and actin were upregulated in *Cucujus clavipes puniceus* (Coleoptera) during the winter, whereas tubulin and tropomyosin were upregulated during the summer, suggesting a seasonal cytoskeleton rearrangement [45]. The high expression of cytoskeleton genes in *T. diversipes* might also be a response to the lower temperatures and/or low humidity in the tropical winter, even though these changes are not as drastic as in temperate zones. However, it is also possible that alterations in the *T. diversipes* cytoskeleton are related to changes in other major pathways.

Cuticle genes have also been reported to be upregulated during diapause in different developmental phases and species [45–47]. This overexpression is generally associated with a thicker cuticle, which prevents water loss [5, 45, 46]. Conversely, during *T. diversipes* diapause, most of the genes related to the cuticle were downregulated. The properties of the insect cuticle vary during development, and the epidermis synthesizes three types of cuticle: larval, pupal, and adult [48]. It is likely that *T. diversipes* has a higher demand for cuticle synthesis during the transition among larval instars than when maintaining the larvae during diapause. Given the fact that each pool of non-diapause larvae was prepared by mixing individuals from different larval instars, it is reasonable to assume that the non-diapause larvae expressed more cuticle genes due to ecdysis. This finding indicates that the dry winter conditions are not the main challenges faced by tropical species undergoing diapause.

#### Sterol and lipid metabolism

*Acyl-CoA binding protein homolog* (*ACBP*) was downregulated in *T. diversipes* diapause, as well as in *H. armigera* [49]. This gene product is involved in the regulation of ecdysteroid biosynthesis [49]. *Acyl-CoA synthetase* and *glycerol 3-phosphate dehydrogenase* genes were also downregulated in *T. diversipes* diapause; these genes are important in the pathways of triacylglycerol and phospholipid precursor production [42]. Triacylglycerol is the main caloric reserve during diapause [50] and phospholipids are membrane components [42]. Thus, the low expression of these genes is expected during diapause because higher amounts of energy storage are produced in the previous larval feeding stages.

*Niemann-Pick type C1* and *epididymal secretory protein E1* (*NPC2*) were also downregulated during *T. diversipes* diapause. Previous studies [51–53] have suggested that *NPC* genes are important for ecdysteroid biosynthesis through the regulation of cholesterol uptake and traffic in the prothoracic gland [53]. In the larval phase, diapause is characterized by a reduction in ecdysteroid production by the prothoracic gland [5] and the low expression of these genes may be directly related to these processes. On the other hand, it has been demonstrated that the *NPC2* gene is negatively regulated by juvenile hormone (JH) [54], which at high levels leads to low levels of *NPC2* expression. This indicates that the JH may be involved in preventing the molting process from larva to pupa in *T. diversipes*, as was reported to occur in diapause larvae of *Diatreae grandiosella* (Lepidoptera) [55].

#### Cell cycle

In general, cells of organisms in diapause do not divide; they remain halted at G0/G1 or G2 phases [36, 56–58]. In agreement with this assumption, the *Rho-related BTB domain-containing protein 2* (*RhoBTB2*) and *SAM/SH3 domain-containing protein 1* (*SASH1*) genes involved in the repression of cell proliferation were upregulated during *T. diversipes* diapause.

It has been demonstrated that *RhoBTB2* expression inhibits cellular proliferation in breast cancer [59] through a downregulation of cyclin D1 [60, 61]. In glioma cell lines, the overexpression of *SASH1* reduces the growth and viability of tumor cells. This effect is related to cell cycle arrest in the G0/G1 phase [62]. Similar to the upregulation of *RhoBTB2,* the upregulation of *SASH1* also reduces the amount of cyclin D1 protein, which is related to a decrease in cell proliferation [62]. The increased expression of these two genes may play an important role in cell cycle arrest during diapause in *T. diversipes*. Though we did not find differential expression in the *cyclin D1* gene, we verified that *cyclin-dependent kinase 7* was downregulated and that *CDK5 regulatory subunit-associated protein 2* was upregulated during diapause. To the best of our knowledge, the present study is the first to report that *RhoBTB2* and *SASH1* have a role in the diapause phenomenon.

#### Immune response

Genes involved in the immune response, such as *toll*, *apidaecin*, and *peptidoglycan-recognition protein SB1* were upregulated during *T. diversipes* diapause. Mutations in the *toll* gene have a direct influence on the survival rate of *Drosophila* infected by fungus [63]. *Apidaecin* and *peptidoglycan-recognition protein SB1* are known to codify antimicrobial peptides [64, 65]. Genes associated with immunity were also upregulated during *Aedes albopictus* [47] and *S. crassipalpis* diapause [33], suggesting that a high investment in defense against pathogens may be a common strategy during this phase [47].

#### Heat shock proteins

Genes codifying for heat shock proteins (HSPs) were among the first to have gained attention and to have been extensively described as associated with diapause [66]. These proteins have been well documented as involved in diapause of several organisms, from fungal dormancy to mammalian hibernation [5].

Most of the genes related to HSPs were downregulated in *T. diversipes* diapause: *60-kDa heat shock protein mitochondrial*, *activator of 90-kDa heat shock protein ATPase homolog 1*, *heat shock 70-kDa protein cognate 4*, *heat shock protein 75-kDa mitochondrial*, and *heat shock protein 81–1*. *HSP* gene expression apparently has a mixed pattern during insect diapause, for example, a HSP60 family member is upregulated during *S. crassipalpis* diapause [67], whereas the opposite was verified for *T. diversipes*. The *hsc70* gene was downregulated in *Culex pipiens* [68] and *T. diversipes*, but was constantly expressed during diapause of *S. crassipalpis* [67], *M. rotundata* [69], *Helicoverpa zea* [70], and *Chilo suppressalis* [71]. It has been suggested that the low expression of *hsc70* is related to long periods of reduced food intake [72], which should be expected during diapause.

Nevertheless, some *HSPs* had conserved levels of expression reported in different species. *Hsp90* was commonly downregulated during diapause of *S. crassipalpis* [67], *H. zea* [70], *H. armigera* pupae [73], and *Nasonia vitripennis* larvae [6]. The downregulation of *hsp90* can be linked to low amounts of ecdysteroids. For example, when ecdysteroids are absent, which is a characteristic of pupal and larval diapause, *hsp90* is downregulated [5]. In *T. diversipes* diapause larvae, this specific gene was not found to be differentially expressed, but interestingly the *activator of 90-kDa heat shock protein ATPase homolog 1* gene was downregulated. This gene is essential to the activation of the HSP90 ATPase [74].

Only one *HSP* gene was upregulated during *T. diversipes* diapause: the *protein lethal (2) essential for life* (*l(2)efl*). This gene codifies for a small heat shock protein (sHsp) from the HSP20 family and it is also upregulated during *Nasonia* diapause [6]. The upregulation of HSPs were already demonstrated to be essential to cold tolerance in *S. crassipalpis* [67]*.* However, the fact that only one *Hsp* gene was upregulated in *T. diversipes* diapause suggests that cold is not a challenge encountered in this phase and the overexpression of this gene might have other, yet non-described functions [5].

#### Circadian clock

There is evidence that circadian clock gene expression does not follow a pattern when comparing diapause of different species. Mutations in *period* (*per*) do not affect the ability of *Chymomyza costata* to enter diapause [5], whereas the knockdown of *per*, *cryptochrome2* (*cry2*), and *timeless* (*tim*) prevent the onset of diapause in *C. pipiens* [75]. In the adult bean bug (*Riptortus pedestris*), the knockdown of *per* and *cry2* leads to ovary development, even under diapause conditions, whereas blocking the *clock* gene suppresses ovarian development [76–78].

In *T. diversipes* diapause, the genes *clock*, *D-site binding protein* (*DBP*), and *Nuclear factor interleukin-3-regulated protein* (*NFIL3*) were all upregulated. It is known that the gene *per* can be positively regulated by the *clock* product [78] and can also be affected by D box genes (*DBP* and *NFIL3*). The D box genes act in an opposite way, where *DBP* activates *per*, *NFIL3* represses it [79]. A more detailed study is needed to elucidate the circadian cycle of *T. diversipes* diapause. Nonetheless, the high expression of *clock*, *DBP*, and *NFLI3* enable us to infer that these genes might be involved in the transcriptional regulation of *per*.

Photoperiod is the main diapause initiating stimulus in organisms from temperate zones, but the mechanisms involved in measuring day length and all the ensuing pathways that produce an endocrinal response remain unsolved [78]. The importance of circadian clock genes in the regulation of photoperiod in many organisms has already been demonstrated [76, 80]. However, in bees, the expression of the clock gene *per* has no variation in response to light [81]. For example, the circadian rhythm of honeybees is an endogenous process that changes with age. Young individuals that perform nest activities have a lower expression of *per* and constant levels of *clock* mRNA during the day ([81], reviewed in [82]). In bumblebees, gynes (virgin queens) emerge from pupae without noticeable circadian rhythm expression, but during adult life, these bees exhibit plasticity in rhythm. Thus, the queens, depending on the presence of her brood, switch their activity and circadian rhythm patterns [83]. Conversely, solitary insects present a defined circadian rhythm as soon as they emerge (reviewed in [76]). The absence of a clear circadian rhythm during the larval stage in some species, and the fact that, in bees, the development of larvae, pupae and even early adult stages, occurs in a sealed nest under complete darkness [11], suggest that the photoperiod is not directly involved in inducing diapause in these insects [84]. Therefore circadian genes here verified as DEGs in *T. diversipes* diapause may be related to other clues or are responses to other stimuli besides light.

#### Epigenetic control

Epigenetic mechanisms, which are heritable changes in gene expression capable of inducing different phenotypes without alterations in the DNA sequence [85], have recently been related to dormancy. It is known that environmental alterations can directly modify epigenetic marks mediating phenotypic plasticity [86]; thus, it is likely that a phenotype highly influenced by environmental conditions, as diapause is, can be epigenetically controlled. A number of epigenetic mechanisms (methylation, histone acetylation, and small regulatory RNAs) have been reported as being related to diapause [87–89].

In *T. diversipes*, the diapause DEGs related to epigenetic factors are primarily involved in histone modifications. One of these genes, *histone acetyltransferase KAT7*, was upregulated during diapause. Histone acetyltransferases acetylate histone cores, resulting in regulatory effects at the chromatin structure level, and consequently, on gene transcription [85]. It has been demonstrated that this specific gene product activates transcription during mouse development [90]. Another upregulated gene that possibly affects histone organization is the *bromodomain adjacent to zinc finger domain protein 2B* (*BAZ2B*). The BAZ2B protein contains a bromodomain, which are protein interaction modules that specifically recognize ε-N-lysine acetylation motifs [91]. Although the functions of the BAZB2 protein have not been described yet, this protein has been demonstrated to interact with histones H1–4, H2A, H2B, H3, and H4, with the strongest interactions occurring with histone H3 [91].

Histone genes were also found to be differentially expressed during *T. diversipes* diapause, with H3 upregulated, whereas H2A and H2B were downregulated. During chromatin remodeling, which may be driven by hyperacetylation, it has been shown that histones can be lost [92, 93]. The findings of the present study thus suggest that chromatin modifications are involved in gene expression changes during diapause. Moreover, the overexpression of *histone acetyltransferase KAT7* and *BAZB2* implies that histone acetylation, in H3 and H4, would represent one of the main mechanisms.

## Conclusion

Diapause is an important and noteworthy phenomenon that has been extensively studied in several species from different taxonomic groups. Particularly in bees, the knowledge of the molecular basis underlying this phenomenon can help to understand other relevant mechanisms of bee evolution, such as the evolution of social behavior [94, 95]. In addition, given its bivoltine behavior, the solitary bee *T. diversipes* is an excellent model for studying diapause in natural conditions and non-temperate areas, where a major knowledge gap is present in diapause studies.

Here, we performed RNA-Seq comparisons and identified that the change in gene expression is launched in the 5th larval instar (larvae collected at the beginning of the dry season); we also identified several DEGs related to diapause in *T. diversipes* larvae. Some of these have already been described as being related to diapause in other organisms; thus our data reinforced the genetic diapause toolkit hypothesis. Moreover, new diapause-related genes were assigned. Among those, some are of particular interest, such as *Niemann-Pick type C1*, previously described as related to the larva dauer formation in *C. elegans*; *NPC2* and *Acyl-CoA binding protein homolog*, which are involved in ecdysteroid synthesis; *RhoBTB2* and *SASH1*, that are associated with the cell cycle; and *Histone acetyltransferase KAT7*, that regulates transcription through epigenetic mechanisms. These are all relevant candidate genes that need to be investigated during diapause of other species.

Our results also add important information to the conserved molecular pathways of diapause and could prove useful in guiding more detailed future studies about diapause in *T. diversipes*. However, further efforts are still required to identify other diapause-related genes and increase database content, towards the goal of gaining a better understanding of the evolutionary conservation across species. Finally, diapause is phenotypically plastic and influenced by external stimuli, thus understanding diapause may help us to elucidate how environmental signals are epigenetically translated, a research field poorly understood to date.

## Additional file

Additional file 1: Tables presenting the differentially expressed transcripts and gene annotation results. (XLSX 413 kb)

## Abbreviations

*ACBP*: *Acyl-CoA binding protein homolog*
*BAZ2B*: *Bromodomain adjacent to zinc finger domain protein 2B*
BUSCO: Benchmarking Universal Single-Copy Orthologs
*DBP*: *D-site binding protein*
DEGs: Differentially expressed genes
G1: First generation
G2: Second generation
GO: Gene Ontology
*GST*: *Glutathione-S-transferase*
HSPs: Heat shock proteins
JH: Juvenile hormone
*NFIL3*: *Nuclear factor interleukin-3-regulated protein*
*NPC1*: *Niemann-Pick type C1*
*NPC2*: *Epididymal secretory protein E1*
*per*: *Period*
*RhoBTB2*: *Rho-related BTB domain-containing protein 2*
*SASH1*: *SAM/SH3 domain-containing protein 1*
TCA: Tricarboxylic acid
